# Soil bacteria and fungi communities are shaped by elevation influences in Colombian forest and páramo natural ecosystems

**DOI:** 10.1007/s10123-023-00392-8

**Published:** 2023-07-17

**Authors:** Glever Alexander Vélez-Martínez, Wendy Lorena Reyes-Ardila, Juan Diego Duque-Zapata, Paula Andrea Rugeles-Silva, Jaime Eduardo Muñoz Flórez, Diana López-Álvarez

**Affiliations:** Grupo de Investigación en Diversidad Biológica, Departamento de Ciencias Biológicas, Facultad de Ciencias Agropecuarias, Universidad Nacional de Colombia-Sede Palmira, Carrera, 32 No. 12-00, 763536 Palmira, Valle del Cauca Colombia

**Keywords:** Metataxonomic, Soil microbial ecology, Colombian natural ecosystems, Altitudinal gradient

## Abstract

The influence of elevation on natural terrestrial ecosystems determines the arrangements of microbial communities in soils to be associated with biotic and abiotic factors. To evaluate changes of fungi and bacteria at the community level along an elevational gradient (between 1000 and 3800 m.a.s.l.), physicochemical measurements of soils, taxonomic identifications of plants, and metabarcoding sequences of the 16S rRNA gene for bacteria and the ITS1 region for fungi were obtained. The bacterial taxonomic composition showed that Acidobacteriota increased in abundance with elevation, while Actinobacteriota and Verrucomicrobiota decreased. Furthermore, Firmicutes and Proteobacteria maintained maximum levels of abundance at intermediate elevations (1200 and 2400 m.a.s.l.). In fungi, Ascomycota was more abundant at higher elevations, Basidiomycota tended to dominate at lower elevations, and Mortierellomycota had a greater presence at intermediate sites. These results correlated with the edaphic parameters of decreasing pH and increasing organic carbon and available nitrogen with elevation. In addition, the Shannon index found a greater diversity in bacteria than fungi, but both showed a unimodal pattern with maximum values in the Andean Forest at 2400 m.a.s.l. Through the microbial characterization of the ecosystems, the elevational gradient, soil properties, and vegetation were found to exert significant effects on microbial communities and alpha diversity indices. We conclude that the most abundant soil microorganisms at the sampling points differed in abundance and diversity according to the variations in factors influencing ecological communities.

**Supplementary Information:**

The online version contains supplementary material available at 10.1007/s10123-023-00392-8.

## Introduction

Natural terrestrial ecosystems such as conserved areas maintain highly stable physical interactions among living beings, which generate ecosystem services without human intervention (Oguh et al. 2021). Their distribution in topographic regions is largely associated with elevation, as distinct elevations imply particular conditions in temperature, precipitation, and soil properties that change the configuration of organisms in ecological communities (Ahmad et al. 2020; Massaccesi et al. 2020; Ramírez et al. 2023). Those changes in soil bacteria and fungi imply specific taxonomic compositions associated with vegetation and parameters such as pH and available organic carbon (Cui et al. 2019; Ji et al. 2022). Natural ecosystems such as forests and páramos can maintain differential microbial ecological structures, which could be performing important local processes including nutrient cycling and assistance in plant nutrition (Hoch et al. 2019; Jiao et al. 2021). In this sense, elevational gradients constitute the most powerful natural laboratories for evaluating ecological hypotheses and understanding the responses of organisms to geophysical influences (Körner 2007).

In recent years, metabarcoding has identified non-culturable species in microbial communities and made inferences regarding soil health through taxonomy (Duque Zapata et al. 2023). With these advances, research has focused on understanding the predominant factors that limit or favor the distribution of microbiomes in ecosystems differing in elevation (Wang et al. 2021; Zhang et al. 2020). However, few such efforts have focused on South American tropical forests and páramos. Across elevational gradients, bacteria and fungi exhibit different behaviors in ecosystems with differential influences of biotic and abiotic parameters. For example, both groups have been shown to decrease in diversity with increasing elevation in the Tibetan Plateau (Shen et al. 2019; Wang et al. 2015), but there is also evidence of maximum diversity at intermediate elevations (Liu et al. 2022; Singh et al. 2012), or no significant variations between sampling points (Fierer et al. 2011; Meng et al. 2013). Therefore, evaluating the composition and diversity of soil microbial communities in different ecosystems must be a priority to maintain genetic pools, monitor alterations in conserved areas, and understand future responses to climate change.

Ultimately, the complex interactions among soil microbial communities in relation to elevation have diverse implications for natural areas and their associated diversity. Colombian ecosystems, including tropical forests and páramos, provide a unique opportunity to investigate such relationships. In this article, our objective was to compare the taxonomic composition and diversity of fungi and bacteria at four sampling points representing three ecosystems in Colombia along an elevational gradient. Additionally, we aimed to evaluate the associations of microbial communities with physicochemical parameters and plant genera, in order to predict the ecological functions of soil communities.

## Materials and methods

### Soil sampling and plant characterization

**Fig. 1:**
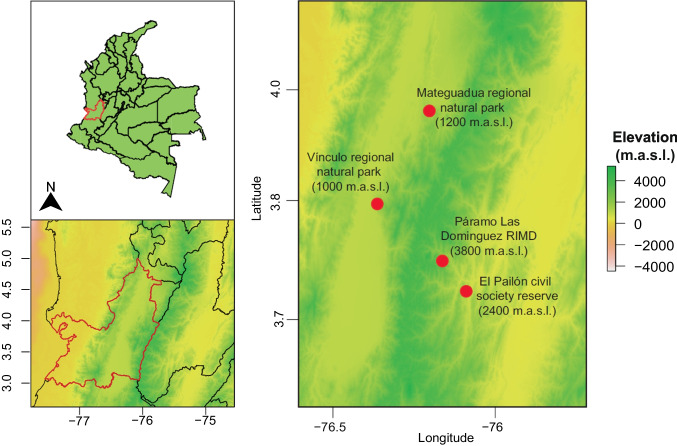
Geographic location of sampling points in the central mountain range of Valle del Cauca, Colombia. RIMD, regional integrated management district

Sampling was conducted between October and December 2021 along an elevational gradient ranging from 1000 to 3800 m.a.s.l. in the Valle del Cauca located in the central range of the Andes, Colombia (Fig. 1). The study sites included El Vínculo Regional Natural Park (tropical dry forest, 1000 m.a.s.l.), Mateguadua Regional Natural Park (tropical dry forest, 1200 m.a.s.l.), El Pailón Civil Society Reserve (Andean Forest, 2400 m.a.s.l.), and Las Domínguez Regional Integrated Management District (páramo, 3800 m.a.s.l.) (Fig. 1). At each site, three 10 m × 10 m plots were established, separated from each other by 20 m. From each plot, three soil samples were collected from the top 25 cm using 15 mL Falcon tubes for microbial analysis, three 1 kg soil samples from the same points for physicochemical analysis, and taxonomic determinations were made for every plant. Microbial analysis samples were stored at −80 °C until DNA extraction.

### Soil physicochemical analysis

For the physicochemical analysis, samples were evaluated by measuring pH, effective cation exchange capacity (ECEC), electrical conductivity (EC), soil organic matter (SOM), soil organic carbon (SOC), available nitrogen (N), available phosphorus (P), available sulfur (S), calcium (Ca), magnesium (Mg), potassium (K), sodium (Na), boron (B), iron (Fe), copper (Cu), manganese (Mn), and zinc (Zn), as well as bulk density (BD) and texture (Tx). Results were analyzed using a non-parametric Kruskal-Wallis test with Jamovi 2.2.5 (The Jamovi Project 2021) and a Pearson correlation heatmap in R version 4.2.2.

### Bacteria and fungi DNA extraction and metabarcoding

DNA from the microorganisms was extracted using the commercial DNeasy PowerSoil Pro Kit (Qiagen, Hilden, Germany) following the manufacturer’s protocol. The quality of the extractions was evaluated using spectrophotometry (Colibri Titertek Berthold 84030) and 0.8% agarose gels. Subsequently, libraries were created for the V3-V4 regions of the 16S rRNA gene for bacteria using the primers 341F CCTAYGGGRBGCASCAG and 805R GGACTACNNGGGTATCTAAT (Hjelmsø et al. 2014) and ITS1 for fungi using the primers ITS5-1737F GGAAGTAAAAGTCGTAACAAGG and ITS2-2043R GCTGCGTTCTTCATCGATGC (Bellemain et al. 2010). Sequencing was performed using Illumina NovaSeq 6000 technology with a depth of ~150,000 reads per sample. Reads from the 36 samples of bacteria and 36 samples of fungi were deposited in the European Nucleotide Archive (ENA) under project number PRJEB61162 and accession numbers ERS14881707 to ERS14881778.

### Bioinformatic and diversity analyses

First, QIIME2 v.2022.2 (Caporaso et al. 2010) was run for quality control, which included filtering, denoise, and discarding chimeras using DADA2 (Callahan et al. 2016). The filtered sequences were taxonomically assigned using the SILVA v13_8 bacterial classifier with 99% similarity and UNITE v. 8-99 for fungi and subsequently normalized to 60,000 reads for bacteria and 80,400 for fungi. Next, the qiime2R v.0.99.6 and phyloseq (McMurdie and Holmes 2013) packages were used in R version 4.2.2 to generate Venn diagrams using the Venn package, taxonomic composition graphs using ggplot2 for the 10 most abundant classifications (phylum, class, and genus), and differential analysis of genera abundances in six paired comparisons between locations using the DESeq2-Bioconductor package (Love et al. 2014). Alpha diversity parameters were subsequently calculated using the microbiome v. 1.18.0-Bioconductor package, including the Shannon diversity index, Berger-Parker dominance, Pielou evenness, abundance-based rarity, and rarefaction richness. Beta diversity was estimated based on the Bray-Curtis distances in a principal coordinate analysis (PCoA) using the phyloseq library (McMurdie and Holmes 2013). Furthermore, multifactorial associations were obtained between microbiome abundances, vegetation, and soil physicochemical measurements using the factoextra package. Finally, the Pearson correlation heatmaps of the most abundant phyla with physicochemical parameters were generated using the MicroViz package (Barnett et al. 2021), and partial least squares path modeling (PLS-PM) graphs were created using the plspm package to relate microbial communities and alpha diversities with altitude, physicochemical measurements, and plant identifications.

### Predictive functional annotations of microorganisms

The reads were functionally annotated by predictive methods using the FAPROTAX program for bacteria (Louca et al. 2016) and the FUNGuild program for fungi (Nguyen et al. 2016). Resulting functional annotations were visualized in a heatmap, as well as through a principal component analysis (PCA) of the categories with the highest number of reads for bacteria, and through functional guild composition graphs for the Ascomycota and Basidiomycota phyla for fungi.

## Results

### Variation in physicochemical parameters

The physicochemical parameters formed two main groups according to the behavior of the measurements across the elevational gradient. The first group consisted of parameters whose mean measurements significantly decreased with elevation, including pH, EC, ECEC, K, Mg, and Ca (Table 1). In contrast, the second group increased their measurements along the gradient from low to high elevations, including SOC, SOM, and N with significant differences between all localities, and Fe with drastic increases in the two highest elevations (Table 1). This resulted in negative correlations with elevation in the first group and positive correlations in the second group (Supplementary Fig. S1). Finally, bulk density (BD) was different only in Pailón, while Na did not show any differences with elevation (Table 1).

**Table 1:** Physicochemical parameters of soil samples collected in southwestern Colombia. Means ± standard deviation; *P*, *p*-value. Different letters in superscript between localities represent a significant difference

Locality	Altitude (m.a.s.l.)	pH	EC (dS/m)	ECEC (cmol+/kg)	SOM (g/100g)	SOC (g/100g)
Vínculo	1000	6.28 ± 0.26^a^	0.38 ± 0.08^a^	37.77 ± 7.17^a^	3.83 ± 0.65^a^	2.22 ± 0.37^a^
Mateguadua	1200	6.57 ± 0.32^a^	0.36 ± 0.03^a^	45.57 ± 4.27^a^	5.34 ± 0.72^b^	3.09 ± 0.42^b^
Pailón	2400	6.12 ± 0.29^a^	1.18 ± 0.71^b^	21.89 ± 7.77^b^	9.69 ± 3.35^c^	5.55 ± 1.94^c^
Domínguez	3800	4.79 ± 0.16^b^	0.47 ± 0.21^c^	8.14 ± 1.72^c^	22.24 ± 4.78^d^	12.79 ± 2.77^d^
*X*^2^		24	19	29	31.24	31.,24
*P*		< .001	< .001	< .001	< .001	< .001
Locality	Altitude (m.a.s.l.)	N (%)	P (mg/kg)	S (mg/kg)	Ca (cmol+/kg)	K (cmol+/kg)
Vínculo	1000	0.19 ± 0.03^a^	5.04 ± 0.47^a^	14.44 ± 4.1^a^	17.28 ± 2.64^bc^	0.57 ± 0.2^a^
Mateguadua	1200	0.26 ± 0.04^b^	6.45 ± 0.38^b^	14.33 ± 4.21^a^	32.76 ± 2.79^a^	0.87 ± 0.18^b^
Pailón	2400	0.48 ± 0.17^c^	6.13 ± 0.66^b^	49.69 ± 53.53^b^	14.87 ± 5.31^c^	0.15 ± 0.04^c^
Domínguez	3800	1.11 ± 0.24^d^	6.11 ± 0.6^b^	8.88 ± 2.63^c^	0.69 ± 0.53^d^	0.29 ± 0.12^d^
*X*^2^		31	20.35	24	30	30
*P*		< .001	< .001	< .001	< .001	< .001
Locality	Altitude (m.a.s.l.)	Na (cmol+/kg)	Mg (cmol+/kg)	B (cmol+/kg)	Fe (cmol+/kg)	Cu (mg/kg)
Vínculo	1000	0.14 ± 0.01^a^	19.79 ± 5.53^a^	0.51 ± 0.1^a^	28.82 ± 7.76^a^	4.23 ± 0.9^a^
Mateguadua	1200	0.15 ± 0.04^a^	11.82 ± 1.77^b^	0.67 ± 0.09^b^	24.53 ± 3.34^a^	3.45 ± 0.85^a^
Pailón	2400	0.16 ± 0.07^a^	6.75 ± 2.83^c^	1.1 ± 0.33^c^	151.94 ± 110.13^b^	3.28 ± 1.01^a^
Domínguez	3800	0.14 ± 0^a^	0.68 ± 0.67^d^	0.14 ± 0.05^d^	515.53 ± 235.85^c^	1.01 ± 0.06^b^
*X*^2^		2	30	29.7	29	22
*P*		0.58	< .001	< .001	< .001	< .001
Locality	Altitude (m.a.s.l.)	Mn (mg/kg)	Zn (mg/kg)	BD (g/cm^3^)	Texture (Tx)	
Vínculo	1000	4.6 ± 1.5^a^	1.01 ± 0.07^a^	1.21 ± 0.12^a^	Clay	
Mateguadua	1200	3.1 ± 0.77^a^	1.08 ± 0.2a^b^	1.06 ± 0.1^a^	Clay	
Pailón	2400	4.06 ± 2.01^a^	1.96 ± 1.24^b^	0.62 ± 0.05^b^	Clay loam	
Domínguez	3800	2.82 ± 2.99^b^	1.56 ± 0.99^ab^	1.06 ± 0.13^a^	Sandy loam	
*X*^2^		13.81	12.52	23		
*P*		0.003	0.006	< .001		

### Analysis of microbial composition

A total of 6,079,109 bacterial reads and 6,164,266 fungal reads were obtained from sequencing, averaging 168,864.13 ± 10,907.52 and 171,229.61 ± 11,376.44 reads per sample, respectively (Supplementary Table S1). After quality control and normalization, 1,941,522 bacterial reads and 2,893,265 fungal reads were retained, averaging 53,931.16 ± 1,339.49 and 80,368 ± 56.58 reads per sample, respectively (Supplementary Tables S2-S3 and Tables S2-S4). A total of 34,437 bacterial ASVs and 25,583 fungal ASVs were identified across all samples (Supplementary Table S3 and Table S4).

**Fig. 2:**
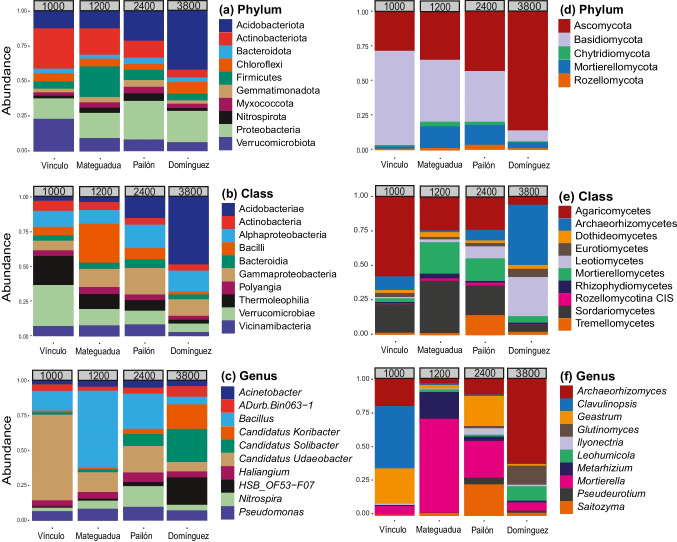
Relative microbial abundances at four sampling points across an elevational gradient in Colombia. Left: bacteria: **a** phylum, **b** class, and **c** genus. Right: fungi: **d** phylum, **e** class, and **f** genus. CIS, class incertae sedis. The gray boxes above each locality indicate the elevation in meters above sea level

The bacterial reads were taxonomically assigned to 32 phyla (100%), 123 classes (95%), 285 orders (90%), 325 families (78%), and 626 genera (72%) (Supplementary Fig. S2 and Table S3). At the phylum level, 100% of taxa were shared across the four localities (Supplementary Fig. S2a and Table S3), with the most abundant being Acidobacteriota (21%), Proteobacteria (20%), Actinobacteriota (15%), Verrucomicrobiota (10%), and Firmicutes (9%) and with lesser abundances of Chloroflexi, Myxococcota, Bacteroidota, Gemmatimonadota, and Nitrospirota (Supplementary Table S3). Across the elevational gradient, Acidobacteriota increased in abundance, from 12% in Vínculo to 43% in Domínguez (Fig. 2a). In contrast, Actinobacteriota and Verrucomicrobiota decreased in abundance, with 23% and 30%, respectively, at the lowest elevation and 6% each at the highest elevation (Fig. 2a). Additionally, the phyla Firmicutes and Proteobacteria had the highest prevalence at intermediate elevations, the former with 23% in Mateguadua and the latter with 28% in Pailón (Fig. 2a). Phyla with low percentages of read assignments did not exhibit a clear behavior across the elevational gradient.

At the class level, 95 classifications (77%) were shared among all locations, with four exclusives to Mateguadua and four exclusives to Pailón (Supplementary Fig. S2b and Table S3). The most abundant class was Acidobacteriae (Acidobacteriota), which showed an increase in abundance across the elevational gradient from 3% at the lowest elevation to 49% in the páramo (Fig. 2b and Supplementary Table S3). Verrucomicrobiae (Verrucomicrobiota) and Thermoleophilia (Actinobacteriota) recorded lower abundances in the páramo (7% and 2%, respectively) compared to the lower elevation tropical dry forest (30% and 22%, respectively) (Fig. 2b and Supplementary Table S3). With maximum percentages at intermediate elevations, Alphaproteobacteria and Gammaproteobacteria had 18% and 20% abundance at Pailón at 2400 m.a.s.l., respectively, while Bacilli (Firmicutes) showed 28% abundance in Mateguadua at 1200 m.a.s.l. (Fig. 2b and Supplementary Table S3).

At the genus level, 274 (44%) genera recorded reads from all localities, with 53 exclusives  from the Pailón forest (Supplementary Fig. S2e). The composition analysis showed that *Candidatus Koribacter* (Acidobacteriae), *Candidatus Solibacter* (Acidobacteriae), and *HSB_OF53-F07* (Ktedonobacteria) increased their abundances along the gradient, from 1 to 20% relative abundance (Fig. 2c and Supplementary Table S3). On the other hand, *Candidatus Udeobacter* (Verrucomicrobiota) dominated relative abundance in Vínculo with 62% and decreased to 7% representativeness in Domínguez (Fig. 2c and Supplementary Table S3). In addition, *Bacillus* (Bacilli) presented maximum percentages at intermediate altitudes, with 56% relative abundance in Mateguadua, as did *Nitrospira* (Nitrospiria) with 13% in Pailón (Fig. 2c and Supplementary Table S3).

On the fungal side, reads were assigned to 7 phyla (59%), 34 classes (49%), 96 orders (45%), 215 families (40%), and 347 genera (36%) (Supplementary Fig. S3 and Table S4). At the phylum level, all 7 classifications were present in the four localities (Supplementary Fig. S3a), with Ascomycota (48%), Basidiomycota (39%), Mortierellomycota (10%), Chytridiomycota (1%), and Rozellomycota (1%) being the most abundant (Fig. 2d and Supplementary Table S4). Across the gradient, Ascomycota dominated at increasing elevations, with percentages ranging from 29% in Vínculo to 86% in Domínguez (Fig. 2d and Supplementary Table S4). In contrast, Basidiomycota decreased in abundance from 68 to 8% with increasing elevations (Fig. 2d and Supplementary Table S4). In addition, Mortierellomycota had an abundance of 15% in Mateguadua and Pailón and 5% in Vínculo and Domínguez. The remaining phyla had around 3% representativeness in the localities and showed no clear trends across the gradient (Fig. 2d and Supplementary Table S4).

At the class level, 24 taxa (70.6%) were recorded from all sampling points, and five were shared among Mateguadua, Pailón, and Domínguez (Supplementary Fig. S3b and Table S4). According to the fungal composition, the ascomycetes that increased in abundance across the elevational gradient were Archaerhizomycetes (from 10 to 44% abundance) and Leotiomycetes (from 1 to 29% abundance), while Sordariomycetes showed the highest abundance in Mateguadua with 39% (Fig. 2e and Supplementary Table S4). In Basidiomycota, Agaricomycetes reported 58% abundance at the lowest elevation and a decrease down to 6% in the páramo. Similarly, Tremellomycetes presented an abundance of 14% in Pailón and 1% in each of the other localities (Fig. 2e and Supplementary Table S4). Finally, the class Mortierellomycetes was the only representative of the Mortierellomycota phylum, with a maximum percentage of an abundance of 23% at Mateguadua, followed by 16% at Pailón (Fig. 2e and Supplementary Table S4).

At the genus level, 141 taxa (41%) were identified and shared across all localities, with 60 exclusives to Pailón and Domínguez, 55 to Mateguadua-Pailón-Domínguez, and 49 to just Domínguez (Supplementary Fig. S3e and Table S4). In terms of composition, *Archaeorhizomyces* (Archaerhizomycetes) maintained increased abundances of Ascomycota, with 20% representativeness in Vínculo and 63% in Domínguez (Fig. 2e and Supplementary Table S4). Additionally, the genera *Glutinomyces* and *Leohumicola* (Leotiomycetes) reported abundances of 2% at lower altitudes and over 10% in the páramo (Fig. 2e and Supplementary Table S4). *Metarhizium* (Sordariomycetes) showed a tendency towards maximum abundance at Mateguadua, with 20% abundance (Fig. 2e and Supplementary Table S4). Regarding Basidiomycota, the genera *Geastrum* and *Clavulinopsis* (Agaricomycetes) reported high abundances and contrasting behaviors across the gradient. *Geastrum* had maximum abundances of 26% and 23% at Vínculo and Pailón, respectively, while *Clavulinopsis* showed a decrease in abundance from 46% in Vínculo to ~1% in each of the other localities (Fig. 2e and Supplementary Table S4). The only genus pertaining to the Mortierellomycota phylum, *Mortierella*, showed dominance at Mateguadua with 69% of the reads from that locality and 28% at Pailón (Fig. 2e and Supplementary Table S4).

### Analyses of alpha and beta diversity

**Fig. 3:**
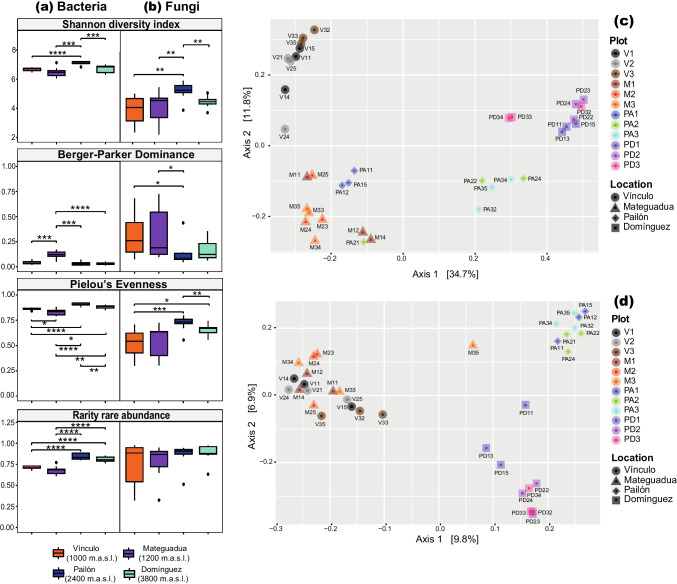
Left: alpha diversity patterns influenced by elevation in (**a**) bacteria and (**b**) fungi. Asterisks denote significant differences between localities: *p < 0.05, **p < 0.01, ***p < 0.001, ****p < 0.0001. Right: principal coordinate analyses (PCoA) with Bray-Curtis distances in (**c**) bacteria and (**d**) fungi. The initials V, M, PA, and PD refer to Vínculo, Mateguadua, Pailón, and Domínguez respectively; the first number is the identifier of the plot and the second of the sample

Alpha diversity was evaluated through measurements of diversity, dominance, evenness, rarity, and richness (Fig. 3 and Supplementary Fig. S4). The Shannon diversity index showed high diversity for both groups of microorganisms (>2) in most of the samples, with bacteria being more diverse than fungi, evidenced by values above 6 (Fig. 3 and Supplementary Table S5). The El Pailón reserve (2400 m.a.s.l.) recorded the highest diversity values of all sites across the elevational gradient, indicating significant differences between the Andean Forest and the other localities (Fig. 3 and Supplementary Table S5).

The results of the Berger-Parker dominance index and Pielou’s evenness index showed the expected inverse relationship. In bacteria, dominance was near zero at all localities, although Mateguadua recorded the least uniform community due to the abundance of *Bacillus*. In fungi, the index showed greater variability in values for the Vínculo and Mateguadua localities, which were statistically different with respect to Pailón, the locality with the lowest dominance (Fig. 3 and Supplementary Table S5). Meanwhile, Pielou’s evenness approached one for bacteria, indicating a proportional distribution of abundances in most taxa, but with differences among localities. In fungi, evenness also showed a greater range of distribution between samples, mainly in Vínculo and Mateguadua (Fig. 3 and Supplementary Table S5). Pailón and Domínguez recorded the lowest values of dominance and the highest values of evenness.

On the other hand, a rarity by abundance was high in the Pailón and Domínguez localities for bacteria, with no differences among the fungal communities. In bacteria, the differences between Vínculo and Mateguadua with respect to Pailón and Domínguez were significant, with a *p*-value < 0.0001 (Fig. 3 and Supplementary Table S5).

Regarding beta diversity, the PCoA plot for bacteria showed a separation of samples according to taxonomic composition by location (Fig. 3c). The first axis, which accounted for 34.7% of the variance, separated samples from Domínguez, together with five samples from El Pailón corresponding to plots 2 and 3, on the positive side of the x-axis (Fig. 3c). The second axis, which accounted for 11.8% of the variance, separated samples from Domínguez and Vínculo on the positive side of the y-axis, and Mateguadua and El Pailón on the negative side (Fig. 3c)). In contrast, the fungal PCoA plot showed a clustering according to ecosystems (Fig. 3d). The first axis, which accounted for 9.8% of the variance, separated most of the samples pertaining to the tropical dry forest on the negative side of the x-axis, while samples from the Andean forests and páramo were recovered on the positive side (Fig. 3d). The second axis, which accounted for 6.9% of the variance, showed a separation between Pailón on the positive side of the *y*-axis and Domínguez on the negative side.

The DESeq analyses found the comparisons with the highest number of differentially abundant genera to be Vínculo vs. Domínguez (47 bacteria and 26 fungi) and Mateguadua vs. Domínguez (26 bacteria and 26 fungi) (Supplementary Fig. S6 and Fig. S7). In bacteria, a higher number of abundant genera from the phyla Acidobacteriota and Proteobacteria were reported in Domínguez, while in Vínculo and Mateguadua, genera pertaining to Actinobacteriota, *Candidatus Udaeobacter* of Verrucomicrobiota, and *Bacillus* of Firmicutes were most abundant, supporting the trends found in the taxonomic composition analysis (Supplementary Fig. S6). In fungi, Ascomycota genera were significantly more abundant in Vínculo and Mateguadua, while they were not necessarily more abundant in Domínguez, as seen in the fungal composition analysis. Meanwhile, Basidiomycota genera followed the general trend of the phylum, showing higher abundances in tropical dry forests (Supplementary Fig. S7).

On the other hand, the comparisons of Pailón vs. Domínguez, Pailón vs. Mateguadua, and Pailón vs. Vínculo revealed 19, 15, and 13 differentially abundant bacterial genera, respectively (Supplementary Fig. S6). In the first comparison, the genera *Candidatus Koribacter*, *Candidatus Solibacter*, and *HSB_OF53-F07* were differentially present in Domínguez, as were *Burkholderia*-*Caballeronia-Paraburkholderia*, *Bacillus*, and *Pedomicrobium* in Pailón (Supplementary Fig. S6c). In the other comparisons, the Acidobacteriota and Proteobacteria genera were most abundant in Pailón, while *Bacillus* and *Candidatus Udaeobacter* were most abundant in Mateguadua and Vínculo, both tropical dry forests. In the fungal comparisons, the contrasts of Pailón vs. Mateguadua, Pailón vs. Vínculo, and Pailón vs. Domínguez showed 19, 23, and 9 differentially abundant genera, respectively (Supplementary Fig. S7). Pailón maintained differences with the other locations through the presence of genera such as *Boletinellus*, *Plectosphaerella*, *Trichoderma*, *Serendipita*, *Geastrum*, and *Glutinomyces*. Furthermore, the tropical dry forests at Mateguadua and Vínculo shared differences with Pailón in genera including *Fusarium*, *Leohumicola*, *Pluetus*, and *Clonostachys* (Supplementary Fig. S7c and Fig. S7d). Domínguez, being a páramo ecosystem, showed differences only in the presence of *Sarcodon* and *Colletotrichum* (Supplementary Fig. S7e).

Ultimately, the Vínculo vs. Mateguadua comparison reported the lowest number of genera with differential abundances, only 10 bacteria and 10 fungi (Supplementary Fig. S6 and Fig. S7). In bacteria, *Bacillus*, *Subdoligranulum*, *Dialister*, *CAG-352*, *HSB_OF52-F07*, and *JG30a−KF−32* were more abundant at Mateguadua, while *Candidatus Udaeobacter* was more abundant at Vínculo (Supplementary Fig. S6F). In fungi, *Trichoderma*, *Geastrum*, *Glutinomyces*, *Beauveria*, and *Mortierella* were more abundant at Mateguadua, while *Clavaria*, *Clavulinopsis*, *Colletotrichum*, *Bionectria*, and *Fusarium* were more abundant at Vínculo (Supplementary Fig. S7F).

### Multifactorial association

The multifactorial analyses revealed relationships between microorganism genera and physicochemical parameters and vegetation in the study locations, where three groups were identified according to the ecosystems (Supplementary Fig. S5 and Table S6). Samples from Vínculo in the tropical dry forest were grouped according to similarities in parameters such as K and Mg, the bacterium *Candidatus Udaeobacter*, the fungi *Clavulinopsis*, *Lyomyces*, and *Apodus*, and six plant taxa including *Anturium*, *Erythroxylum*, and *Monstera* (Supplementary Fig. S5 and Table S6). In Mateguadua, samples were grouped through similarities in parameters such as pH, Ca, Cu, ECEC, Mg, and Mn, along with six bacteria including *Bacillus*, seven fungi including *Metarhizium* and *Fusarium*, and six plant genera including *Heliconia*, *Piper*, and *Eugenia* (Supplementary Fig. S5, Table S1 and Table S6).

In the Andean Forest of Pailón, chemical variables such as B, S, Ca, EC, and Zn were associated, along with seven bacterial genera including *Nitrospira*, *Flavobacterium*, and *Sulfurifustis* and eight plant genera such as the wax palm (*Ceroxylon*), *Xanthosoma*, and *Solanum* (Supplementary Fig. S5, Table S1, and Table S6). Meanwhile, samples from the páramo of Domínguez were grouped together by similarities in 18 bacterial genera, including *Candidatus Koribacter*, *Candidatus Solibacter*, and *HSB_OF53-F07*, along with measurements of organic carbon, organic matter, nitrogen, and iron, and characteristic plants of these ecosystems such as frailejones (*Espeletia*) and clubmosses (*Lycopodium*) (Supplementary Fig. S5, Table S1, and Table S6).

### Predictive functional annotations

**Fig. 4:**
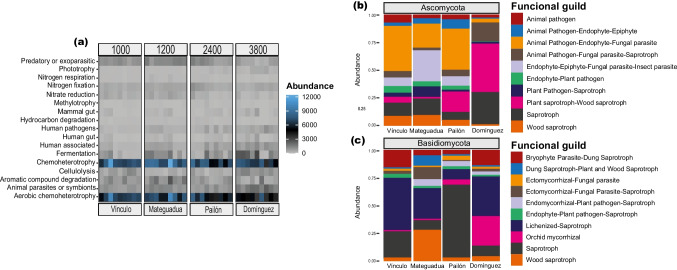
**a** Functional annotations for bacteria, as well as predictive functional guilds for the fungal phyla **b** Ascomycota and **c** Basidiomycota

Seventeen dominant potential functions were annotated in bacteria based on taxonomic identifications using FAPROTAX (Fig. 4a and Supplementary Table S7). The heatmap showed that the chemoheterotrophic and aerobic chemoheterotrophic functional groups had a higher representation of reads at lower elevational locations, with a decrease along the gradient as elevations increased (Fig. 4a and Supplementary Table S7). Nine phyla were found characterized as chemoheterotrophs, with Proteobacteria (55%), Actinobacteriota (28%), and Firmicutes (9%) comprising most of these reads. Within Proteobacteria, the genera *Pseudomonas*, *Acinetobacter*, *Bradyrhizobium*, and *Pedomicrobium* were highly abundant, comprising 76% of the reads in descending order of abundance. Within Actinobacteriota, 70% of the assignments were distributed among the taxa *Acidothermus*, *Streptomyces*, *Mycobacterium*, and *Solirubrobacter*, while in Firmicutes, 48% of the assignments were distributed among *Faecalibacterium*, *Paenibacillus*, *Subdoligranulum*, and *Roseburia* (Supplementary Table S7).

In contrast, phototrophic and fermenting taxa showed an increase in assigned reads along the elevational gradient, with an abundance of *Rhodomicrobium* and *Rhodoplanes* being associated with phototrophy, which predominated in the high-elevation páramo ecosystem of Domínguez (Fig. 4a and Supplementary Table S7). At the same time, several other functions remained unchanged in the number of associated reads along the gradient (Fig. 4a). Additionally, the PCA grouped the functions of chemoheterotrophy, nitrogen cycle, compound degradation, and chitin lysis to the samples from Mateguadua, Vínculo, and four Pailón samples on the positive axis of the abscissa. Interactions with mammals, animal symbionts or parasites, fermentation, phototrophy, and cellulolysis were related to the Domínguez and other Pailón samples (Supplementary Fig. S8).

Within Ascomycota, 10 predictive functional guilds stood out as the most abundant, with four of them assigned as animal pathogens, three as saprophytic, two as endophytic, and one as plant pathogen (Fig. 4b and Supplementary Table S8). Within the pathogens, the animal pathogen-endophyte-fungal parasite guild was highly abundant in Vínculo and Pailón (42% and 38%, respectively), with differentially abundant genera such as *Ilyonectria*, *Fusarium*, and *Volutella* (Fig. 4b and Supplementary Table S8). Within the group characterized by saprophytic functions, the plant-wood saprotroph guild increased its representation along the elevational gradient, from an abundance of 3% in Vínculo up to 45% in Domínguez, in response to the abundance of the genera *Glutinomyces* and *Pseuderotium*. Finally, the endophyte-epiphyte-fungal parasite-insect guild reported the highest number of reads at 1200 m.a.s.l., showing 10% abundance in Vínculo, 29% in Mateguadua, 9% in Pailón, and 1% in Domínguez, following the same trend as *Metarhizium*.

In Basidiomycota, four out of the 10 functional guilds were predominantly mycorrhizal, followed by three saprotrophic guilds, one bryophyte parasitic, one endophytic, and one fungal lichenized guild (Fig. 4c and Supplementary Table S8). Among mycorrhizal guilds, ectomycorrhizal-fungal parasite-saprotroph with the characteristic genera *Clitopilus* and *Entoloma* showed the highest abundance with 11% in Mateguadua (Fig. 4c and Supplementary Table S8). In contrast, orchid mycorrhizae, represented by the genus *Serendipita*, showed 26% abundance in Domínguez, 3% in Pailón, and low representation in tropical dry forests. In addition, the saprotrophic guild showed a high dominance of assignments in Pailón with 67% of the reads pertaining to the genera *Geastrum* and *Leucoagaricus*. Similarly, *Oxyporus* pertaining to the wood saprotrophs and *Coprinopsis* pertaining to dung-plant-wood saprotrophs showed abundances of 28% and 8%, respectively, in Mateguadua and percentages below 2% in the other localities (Fig. 4c and Supplementary Table S8). Finally, the lichenized-saprotrophic fungi guild represented by the genus *Clavulinopsis*, along with bryophytes-saprotrophs dung parasites, had maximum representation in Vínculo, with 48% and 37%, respectively, followed by Domínguez, with 16% and 14%, respectively (Fig. 4c and Supplementary Table S8).

### Relationships of soil microbiomes with environmental measurements

**Fig. 5:**
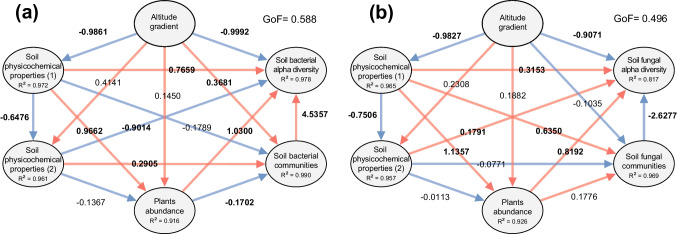
PLS-PM showing the relationships between the elevational gradient, the soil physicochemical properties that decreased along the gradient (1) (pH, ECEC, EC, B, Ca, Mg, K, Na, Cu, and BD), the physicochemical properties that increased along the gradient (2) (SOM, SOC, N, Fe, P, and Zn), the abundance of plant genera, microbial communities, and alpha diversity measurements for **a** bacteria and **b** fungi. The explained variability (*R*^2^) in each grouping and the goodness of fit (GoF) are presented in the graph. The red and blue arrows show positive and negative effects, respectively. The bold partial regression coefficients represent statistical differences with a *p*-value < 0.05

The correlation heatmap showed significant differences between the most abundant phyla and the physicochemical properties of the soil (Supplementary Fig. S9). Overall, Acidobacteriota, Proteobacteria, and Ascomycota were positively and significantly correlated with altitude, SOM, SOC, N, and Fe, while Verrucomicrobiota, Actinobacteriota, and Basidiomycota were correlated with pH, Mg, ECEC, Cu, Mn, and BD. Meanwhile, the PLS-PM showed that the elevational gradient had a negative influence on the first group of physicochemical soil properties (pH, ECEC, EC, B, Ca, Mg, K, Na, Cu, and BD) and that both factors had at least one significant effect on the microbial community or alpha diversity measurements (Fig. 5). In addition, the second group of physicochemical measurements (SOM, SOC, N, Fe, P, and Zn) and the plants did not show significant effects due to elevation, but they did influence the soil microorganisms (Fig. 5).

## Discussion

### Taxonomic composition of the soil microbiome

Overall, 32 bacterial phyla were found to be shared across the four study sites, with Acidobacteriota, Proteobacteria, Actinobacteriota, Verrucomicrobiota, and Firmicutes being the most abundant. Acidobacteriota ranked first in overall abundance, with consecutive increases in reads along the elevational gradient (from low to high elevation), mainly due to the presence of the *Candidatus Koribacter* and *Candidatus Solibacter* genera. This phylum has been reported to reach up to 52% abundance on some surfaces and an average of 20% across various soil environments (Dunbar et al. 2002; Janssen 2006). Although bacterial ecology has associated this phylum with oligotrophic strategies, recent findings suggest contrasting results in its behavior (Kielak et al. 2016). In this study, the maximum abundance was found where organic carbon had the highest measurement, which agrees with positive correlations found between the phylum’s abundance and carbon (Fig. S9) in agricultural soils in the Amazon rainforest (Navarrete et al. 2013) and in a tropical savanna (Pessoa-Filho et al. 2015).

The second-most abundant bacterial phylum, Proteobacteria, was represented by two main classes that showed similar behaviors across the elevational gradient. The trends of Alphaproteobacteria and Gammaproteobacteria in this study are consistent with reports of copiotrophic strategies across an elevational gradient, with a preference for abundances in contrasting ecosystems with wide differences in percentages of carbon and organic matter (Cleveland et al., 2007; Janssen et al., 2002).

Actinobacteriota and Verrucomicrobiota, on the other hand, showed a decrease in abundance across the elevational gradient, and Firmicutes dominated in Mateguadua. Actinobacteriota showed a negative correlation with elevation and a positive correlation with pH (Fig. S9), as reported previously in other studies (Siles and Margesin 2016). Their abundances have also been influenced by the plasticity in their usage of carbon sources, metabolizing everything from fresh substrates such as cellulose up to highly complex ones such as polycyclic aromatics (Huang et al. 2018; Morrissey et al. 2017). The trend of Verrucomicrobiota agrees with the oligotrophic strategy speculated by Brewer et al. (2016) from the genome, with the metabolization of amino acids and substrate-derived vitamins as alternative metabolic pathways. Moreover, the higher abundances at neutral pH values are in accordance with Shen et al. (2017), but contrary to the reported preference for high abundances in acidic soils by Willms et al. (2021). In the case of Mateguadua, the Firmicutes genus *Bacillus* was predominant, possibly due to the influx of labile carbon substrates (Cleveland et al. 2007; Cui et al. 2023) obtained mainly from plant-microorganism associations through rhizodeposition (Romaniuk et al. 2021).

The most abundant fungal phyla in the four study locations were Ascomycota, Basidiomycota, and Mortierellomycota, which together accounted for 97% of the total assignments. Concordant to these findings, Ascomycota and Basidiomycota were also predominant in the soils of the Yungas region in the Andes Mountains in Argentina, across an elevational gradient ranging from 400 to 3000 m.a.s.l. that consisted of Piemonte forests, montane forests, and cloud forests (Geml et al. 2014). These biogeographic variations in fungi with elevation are mainly attributable to climatic factors in different ecoregions, according to the meta-analysis conducted by Větrovský et al. (2019).

The phylum Ascomycota showed increases in its abundance in response to high-elevation conditions (Fig. 2a and Supplementary Fig. S9). This is congruent with the 90% dominance of Ascomycota reported by Bayranvand et al. (2021) at the highest elevation in a gradient from 0 and 2500 m.a.s.l.. Within Ascomycota, Archaeorhizomycetes exhibited a unimodal abundance behavior with respect to elevation, in which edaphic environmental factors such as available carbon, organic carbon, nitrogen, aluminum oxide, and phyllosilicates all contribute to their abundance levels (Pinto-Figueroa et al. 2019). Additionally, abundances of the genera *Glutinomyces* and *Leohumicola*, which show trends of high abundances in the páramos, might also be favored by the maximally elevated values of organic matter in that locality, considering that they were assigned to saprotrophic guilds.

The second most abundant fungal phylum, Basidiomycota, showed decreases its relative abundance with elevation, which is in line with the fungal characterization of beech forests at 1500 m.a.s.l. in Iran (Bayranvand et al. 2021). Along the gradient, the class Agaricomycetes and more specifically the genera *Clavulinopsis* and *Geastrum* were more abundant at 1000 m.a.s.l., possibly due to their ability to break down complex components of lignocellulose in soils, which are mainly found in vegetation at low elevation ecosystems (Lundell et al. 2010).

Lastly, the Mortierellomycota genus *Mortierella* comprised more than 60% of the reads from the most abundant genera in Mateguadua. Previous studies of this phylum have shown correlations with the degradation of lignin and carboxylic-rich alicyclic molecules, with the latter being one of the most recalcitrant compounds in soils (Y. Zhang et al. 2022). Although the genus was more abundant at 1200 m.a.s.l., DESeq analyses (Fig. S7) showed ASVs with differential abundances in Domínguez (3800 m.a.s.l.) and Pailón (2400 m.a.s.l.), suggesting that the reads from these localities could be associated with unknown soil factors that differ from those at lower altitudes.

### Microorganism diversity patterns

The pattern of microbial diversity found in this study showed maximum values of the Shannon index in the Andean Forest ecosystem, at 2400 m.a.s.l. in the El Pailón reserve (Fig. 3). This result of the highest fungal and bacterial diversity found at intermediate elevations contrasts with the typical decrease in diversity along an elevational gradient observed in most macroorganisms (J. Wang et al. 2011). Nevertheless, the trend reported here is consistent with previous research showing bacterial diversity peaks at intermediate elevations (Ren et al. 2018; Shen et al. 2015; Singh et al. 2012), with this behavior being described in the literature as unimodal or inverted U-shaped.

However, some studies have reported patterns in the alpha diversity of fungi and bacteria across an elevational gradient that contrasts with the unimodal trend (Ren et al. 2018; Yang et al. 2022). For example, Ren et al. (2018) found that the evaluated elevational gradient significantly affected bacterial diversity due to changes imposed on plant diversity, soil organic carbon, and total nitrogen but observed no significant effects on fungi. These findings are consistent with quantitative analyses performed by Rahbek (2005), which revealed that the unimodal pattern of elevational richness was the most frequently observed pattern in a review of studies that used large-scale gradients, with 50%, followed by 25% of studies showing a monotonic decreasing pattern.

### Associations of microorganisms with physicochemical parameters and vegetation

In the multifactorial analyses, some associations between physicochemical parameters, vegetation, and fungal and bacterial genera were identified (Supplementary Fig. S5). Parameters such as organic carbon, organic matter, iron, and nitrogen were significantly higher in páramo ecosystems due to their low temperatures, low microbial activity, and possibly high iron concentrations in plant material or erosion processes (C. Liang et al. 2017; Ramírez et al. 2010). Additionally, plants of the *Espeletia* genus (frailejones) have been shown to harbor high abundances of the bacteria *Candidatus Koribacter* and *Candidatus Solibacter* in their roots (Ruiz-Pérez et al. 2016), which break down starch, hemicellulose, pectin, and other compounds (Rawat et al. 2012), possibly explaining the high abundance of these genera in the ecosystem. Meanwhile, *Bejaria* and *Vaccinium* plants of the Ericaceae family have a symbiotic relationship with soil mycorrhizae that aid in the absorption of nutrients from recalcitrant molecules (Flores et al. 2022). Similarly, fungi in the *Archaeorhizomyces* genus have been preliminarily associated with the rhizosphere of tundra and páramo plants, possibly performing bioweathering processes (Pinto-Figueroa et al. 2019).

The Andean Forest was found to be associated with parameters such as B, S, EC, Zn, and P, which recorded maximum measurements at Pailón, 2400 m.a.s.l. (Table 1). Plant associations for this ecosystem include the genus *Myrcia*, which is associated with endophytes that produce metabolites with antimicrobial activity to control bacterial and fungal pathogens (dos Banhos et al. 2014), as well as *Inga*, which similarly associates with bacteria that have the potential to fix nitrogen and degrade lignin in the soil (Eaton and Hamilton 2022). Also, ammonium oxidation processes could be favored by *Nitrospira* bacteria (Mehrani et al. 2020) and nitrogen fixation by *Flavobacterium* (Giri and Pati, 2004).

Finally, the tropical dry forest was associated with chemical variables that were negatively correlated with elevation, such as pH, ECEC, Ca, Cu, Mg, Mn, and K, along with characteristic plants of this ecosystem, such as *Anthurium*, *Heliconia*, and *Piper*. In the climatic conditions of this ecosystem, plants tend to establish symbiotic relationships with microorganisms to improve their nutrition processes and defend themselves against pathogens. Such is the case for *Anthurium*, which is colonized by endophytic fungi during the critical period of propagation, promoting growth and conferring resistance to diseases (Lin et al. 2019). Bacteria found to be associated with this ecosystem include *Bacillus* and *Candidatus Udaeobacter*. The former produces plant growth factors, fixes nitrogen, and solubilizes phosphates (Patiño-Torres and Sanclemente-Reyes 2014), while the latter metabolizes antibiotics in roots (Willms et al. 2020). The associated fungi *Metarhizium* acts as an entomopathogen, saprotrophic, and beneficial endophyte in roots, with the ability to switch between these different lifestyles (St. Leger and Wang 2020).

### Predictive functional annotation of the microbiome

The functional annotation of bacteria found the functions with the highest number of reads to be chemoheterotrophy and aerobic chemoheterotrophy, which were more abundant in Vínculo (1000 m.a.s.l.) and Mateguadua (1200 m.a.s.l.). This is consistent with the results from Liang et al. (2020), in which chemoorganotrophy was found to be predominant but differed between the types of ecosystems studied, noting that this variation was mainly attributed to differences in vegetation among natural systems.

Another pattern within bacterial functions was the increase in abundance of phototrophs across the gradient, specifically pertaining to the genera *Rhodoplanes* and *Rhodomicrobium*. These taxa may be responding to the behaviors of carbon, organic matter, and total nitrogen (Viitamäki et al. 2022).

Finally, the findings related to the fungal functional guilds revealed that functionalities are potentially differentially distributed between the Ascomycota and Basidiomycota phyla. This is like the results from Ren et al. (2021) which studied natural ecosystems across an elevational gradient in the Taibai Mountain range in the Qinling Mountains, China. Basidiomycota was mainly associated with ectomycorrhizal guilds, while Ascomycota was associated with saprotrophic guilds of organic matter and wood.

## Conclusions

This study presented a taxonomic characterization of fungi and bacteria along an elevational gradient spanning three conserved ecosystems in Colombia. The taxonomic composition predominantly found bacteria from the phyla Acidobacteriota, Proteobacteria, Actinobacteriota, Verrucomicrobiota, and Firmicutes and fungi from Ascomycota, Basidiomycota, and Mortierellomycota. The composition varied mostly with factors influenced by the elevational gradient such as pH and organic carbon. The alpha diversity showed a unimodal trend, with the Andean Forest site at an intermediate elevation (Pailón, 2400 m.a.s.l.) recording the highest diversity in both groups. Finally, the elevational gradient had significant effects on the microbial communities and alpha diversity indices, as well as the physicochemical properties and vegetation of the ecosystems. Future research should focus on characterizing the responses of microbial communities in different climatic regimes and their ecological roles in soils.

### Supplementary information

ESM 1

## Data Availability

The sequences of 72 samples have been deposited in the European Nucleotide Archive (ENA) under project number PRJEB61162 and accession numbers ERS14881707 to ERS14881778.
