# Supplementary figures and images for: Soil bacteria and fungi communities are shaped by elevation influences in Colombian forest and páramo natural ecosystems
Source: Int Microbiol. 2023 Jul 17;27(2):377–91. doi: 10.1007/s10123-023-00392-8 (PMC10991037; doi:10.1007/s10123-023-00392-8)

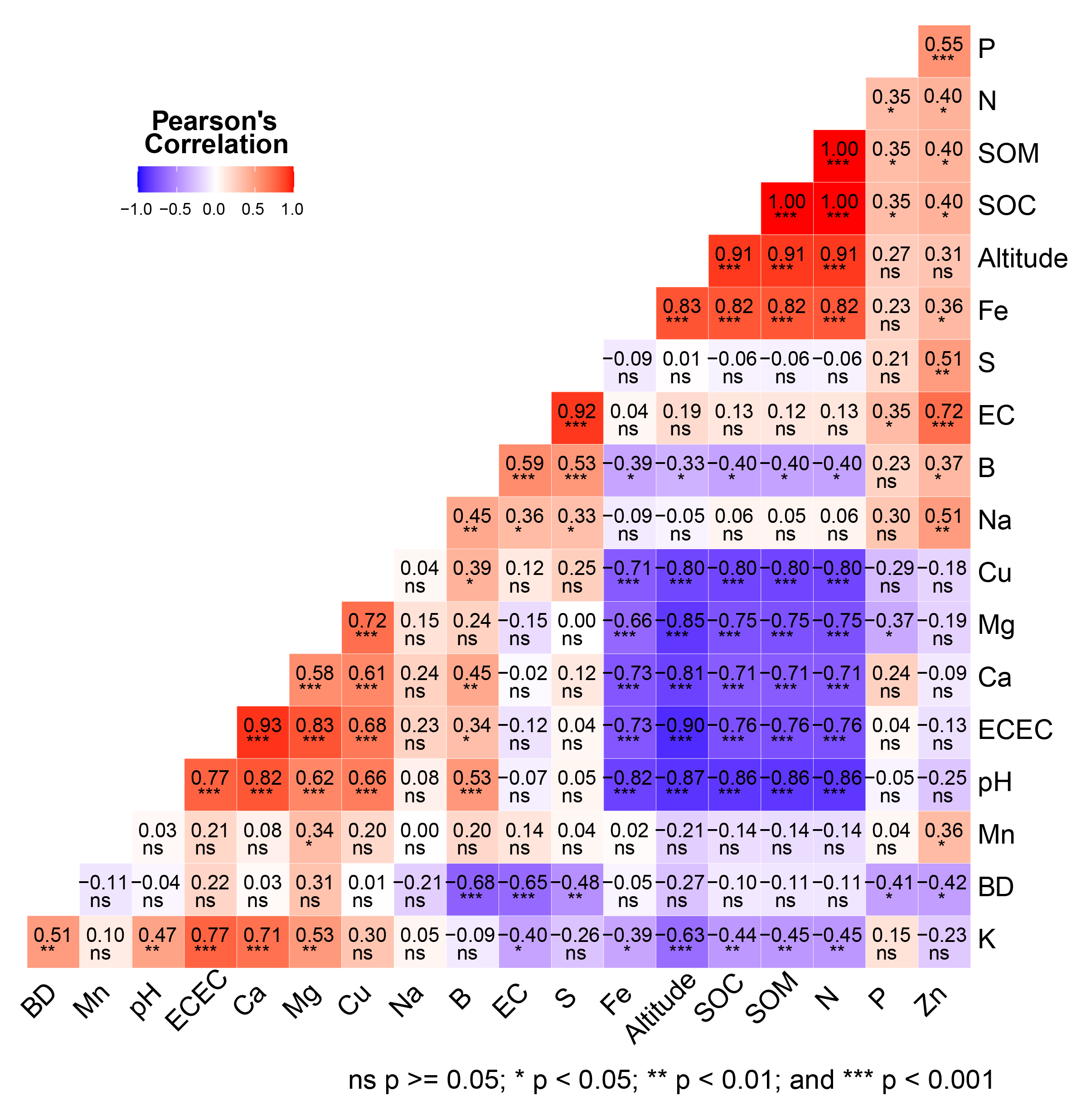

Supplement: Supplementary file 1 — ESM 1 [file 10123_2023_392_MOESM1_ESM.zip › Supplementary Figure S1.png]

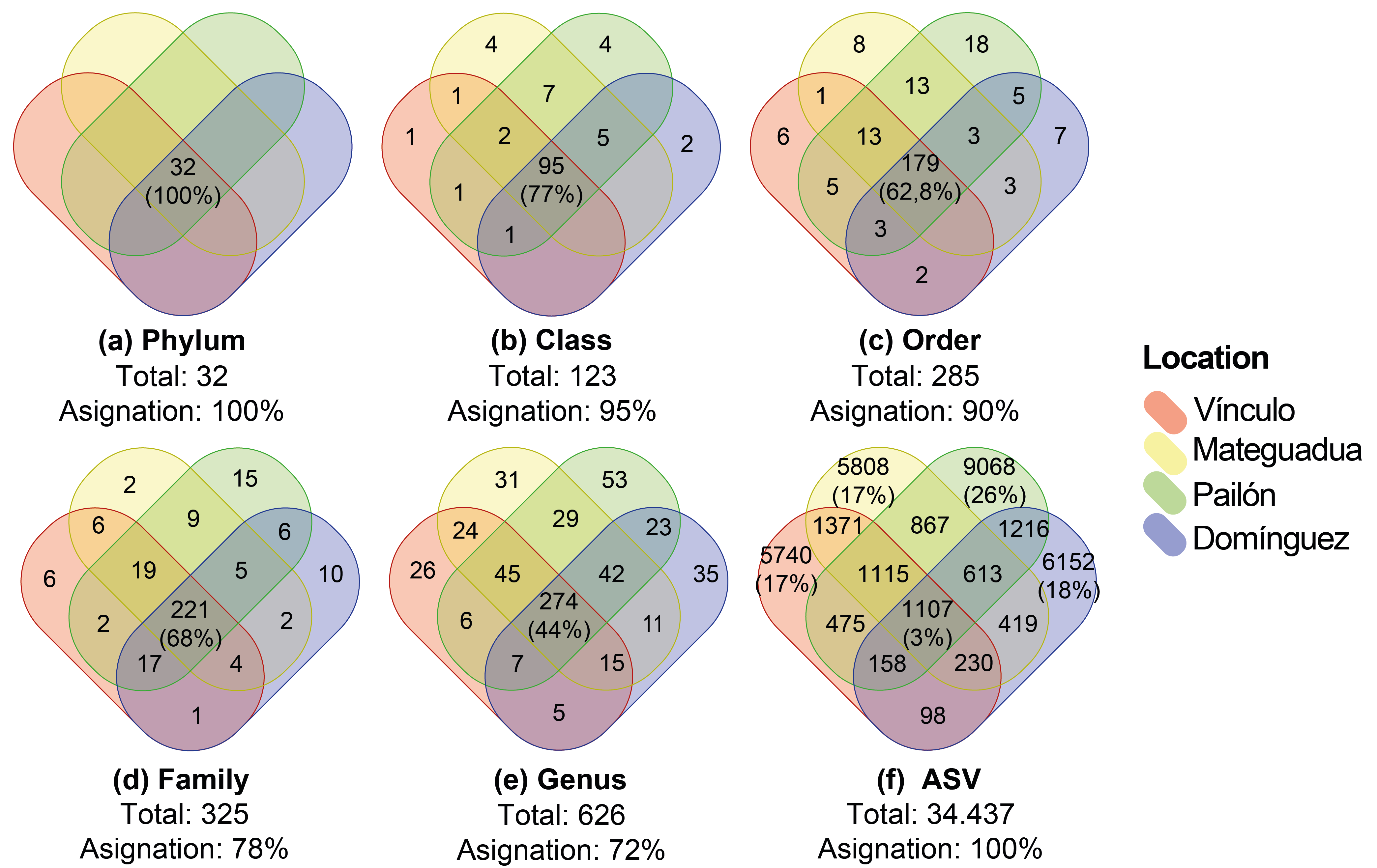

Supplement: Supplementary file 1 — ESM 1 [file 10123_2023_392_MOESM1_ESM.zip › Supplementary Figure S2.png]

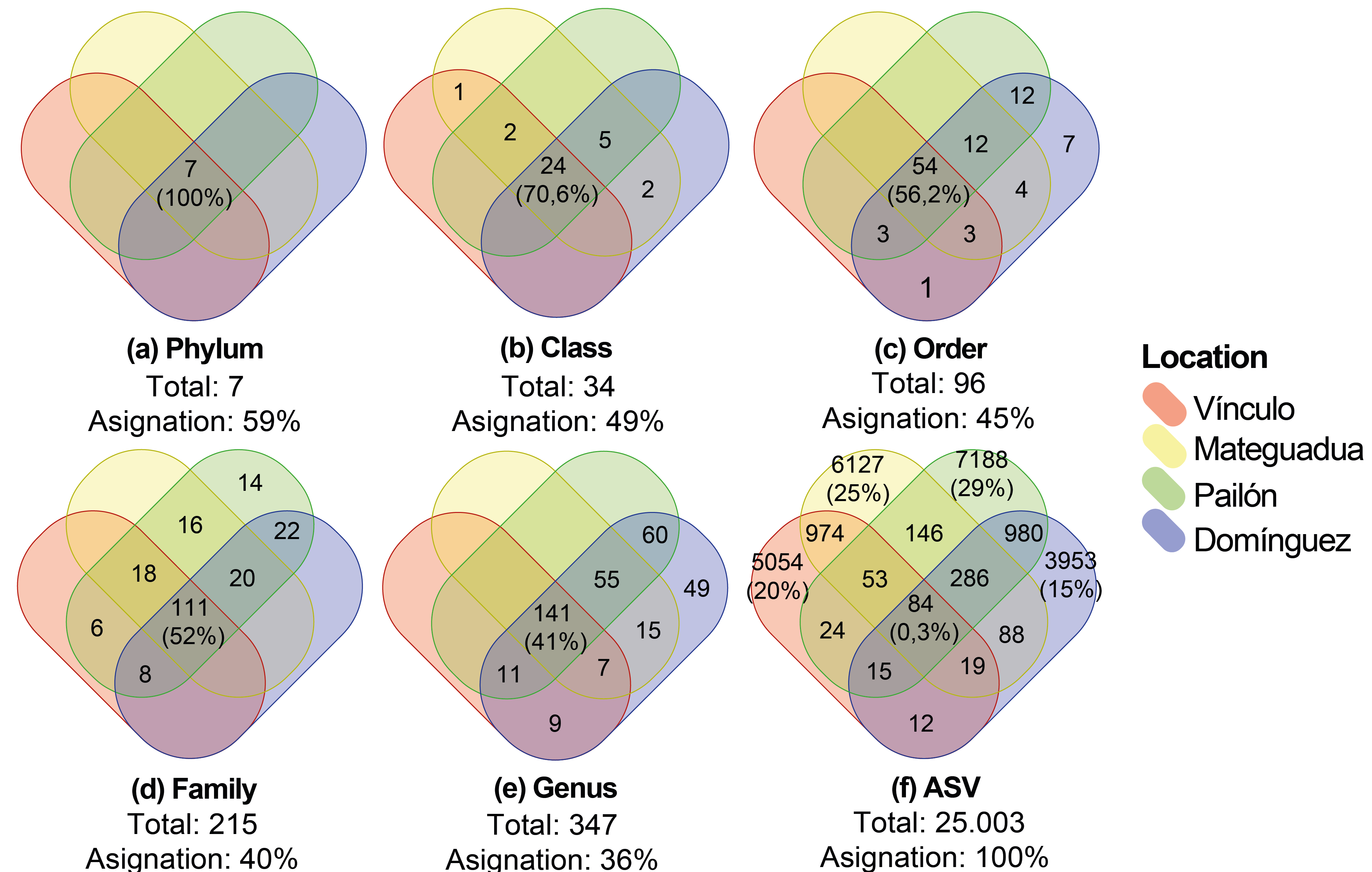

Supplement: Supplementary file 1 — ESM 1 [file 10123_2023_392_MOESM1_ESM.zip › Supplementary Figure S3.png]

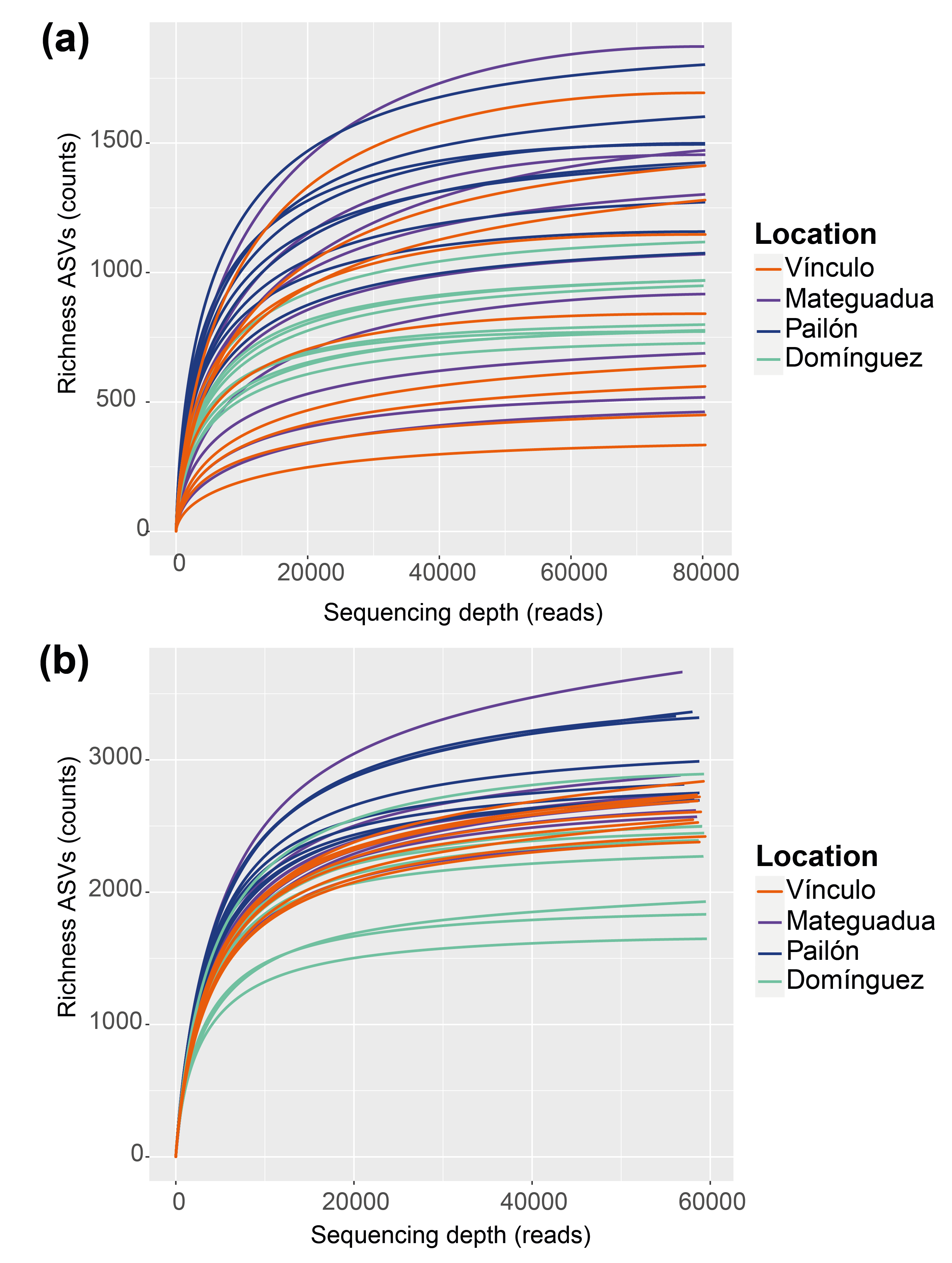

Supplement: Supplementary file 1 — ESM 1 [file 10123_2023_392_MOESM1_ESM.zip › Supplementary Figure S4.png]

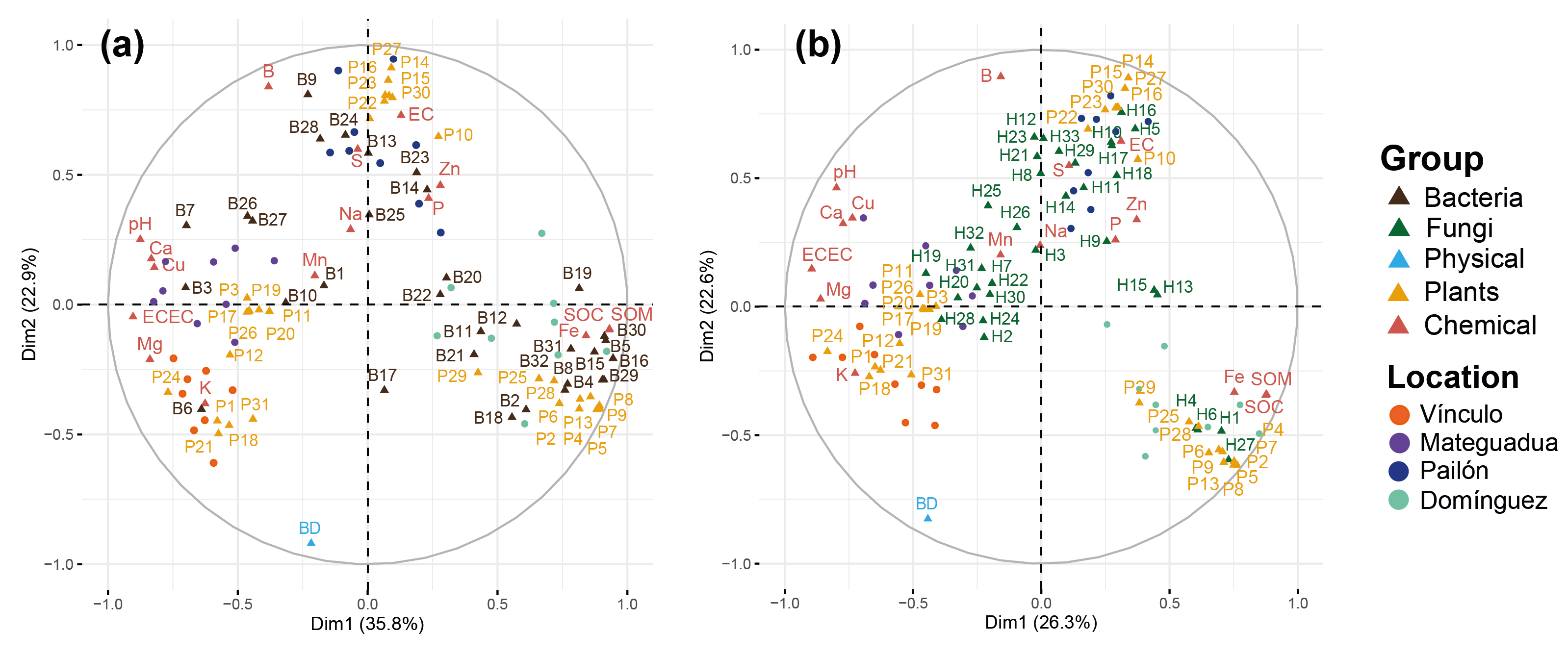

Supplement: Supplementary file 1 — ESM 1 [file 10123_2023_392_MOESM1_ESM.zip › Supplementary Figure S5.png]

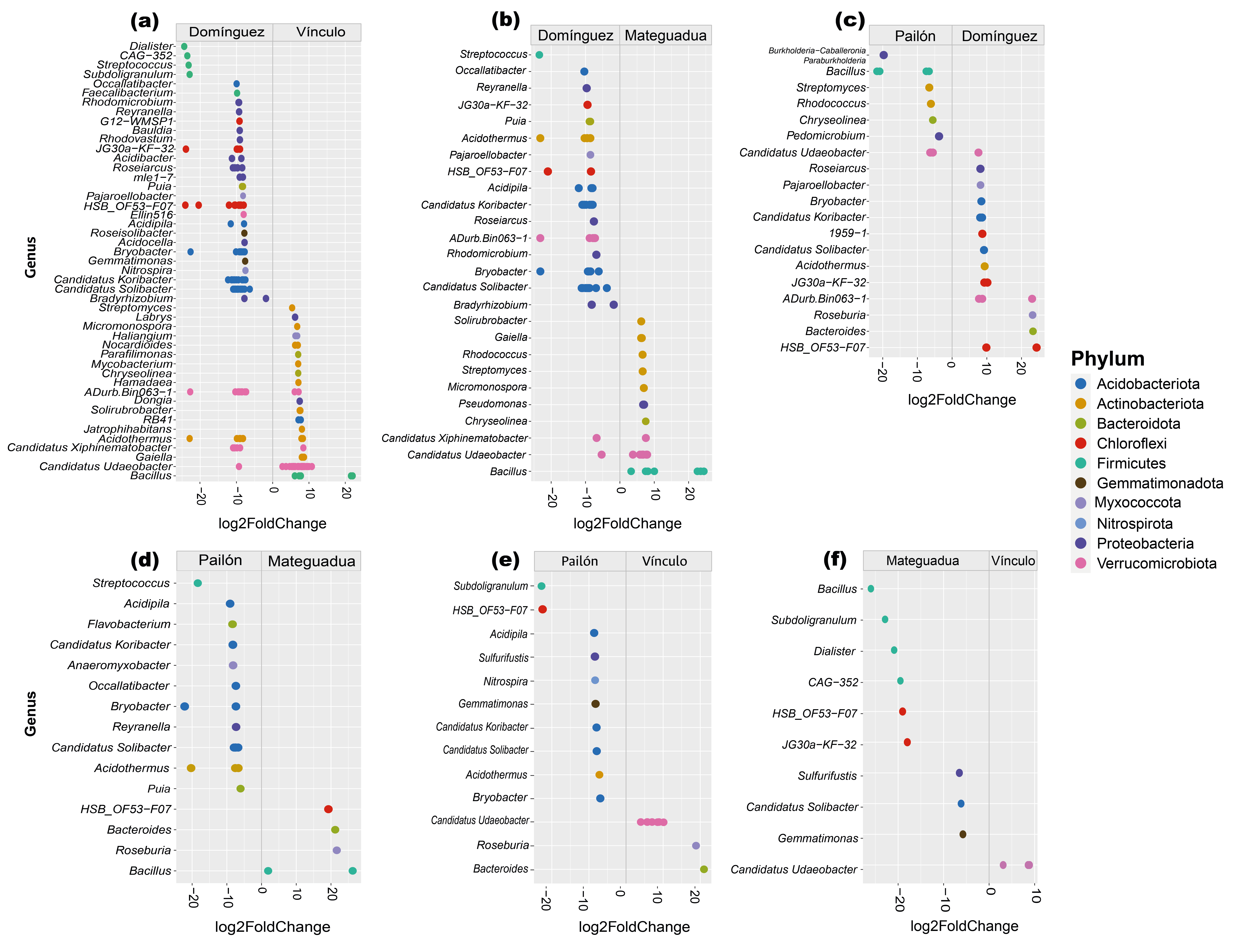

Supplement: Supplementary file 1 — ESM 1 [file 10123_2023_392_MOESM1_ESM.zip › Supplementary figure S6.png]

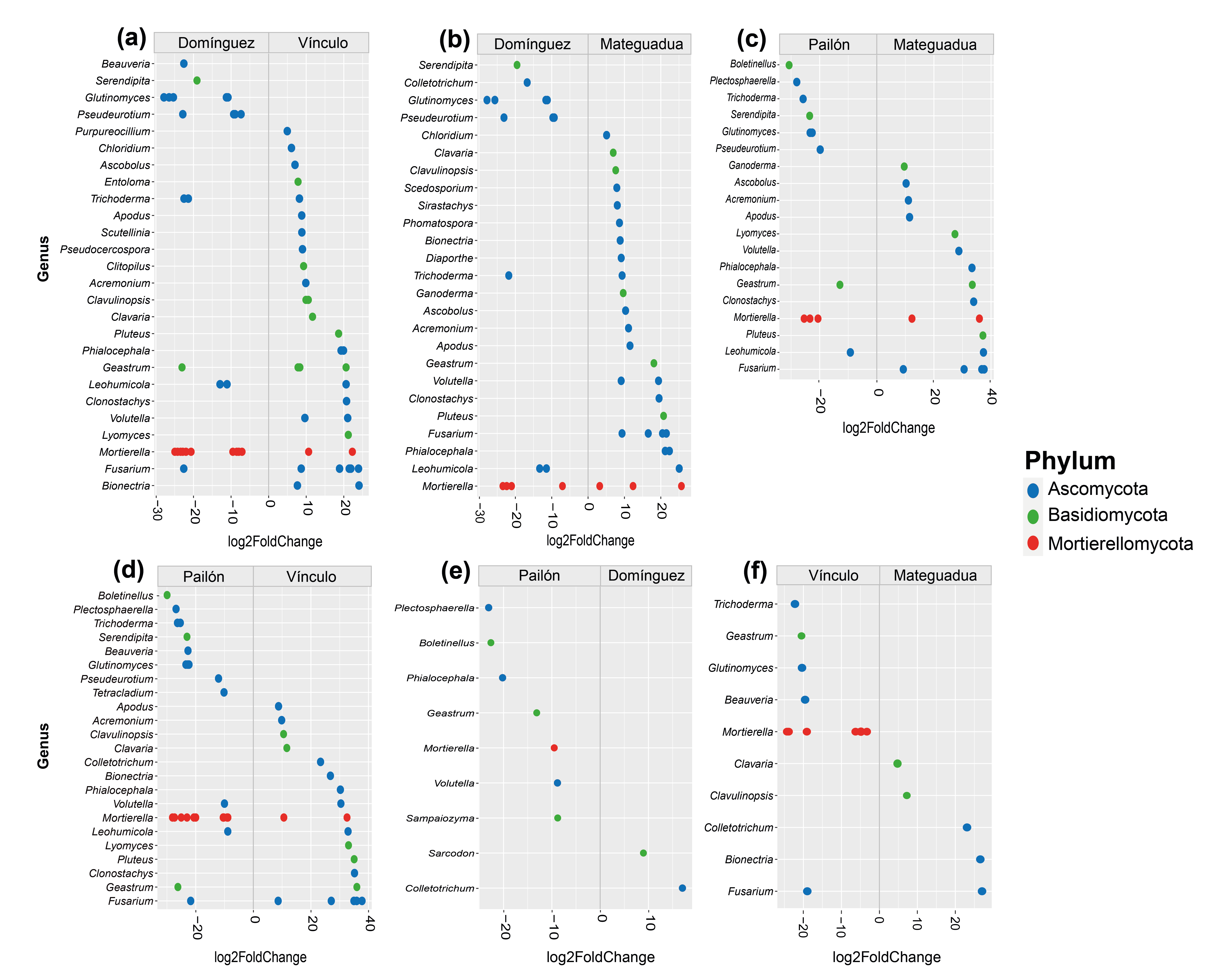

Supplement: Supplementary file 1 — ESM 1 [file 10123_2023_392_MOESM1_ESM.zip › Supplementary Figure S7.png]

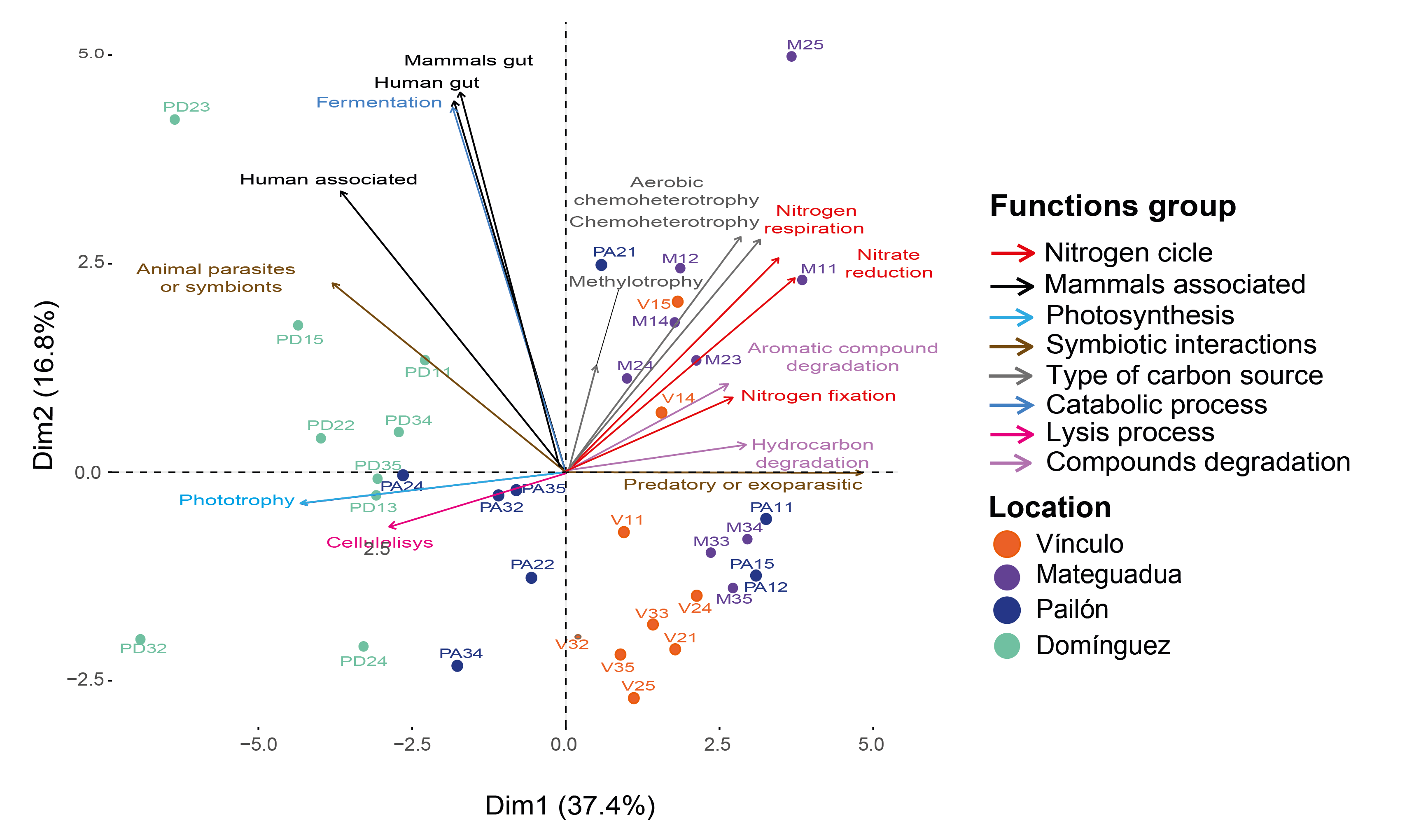

Supplement: Supplementary file 1 — ESM 1 [file 10123_2023_392_MOESM1_ESM.zip › Supplementary Figure S8.png]

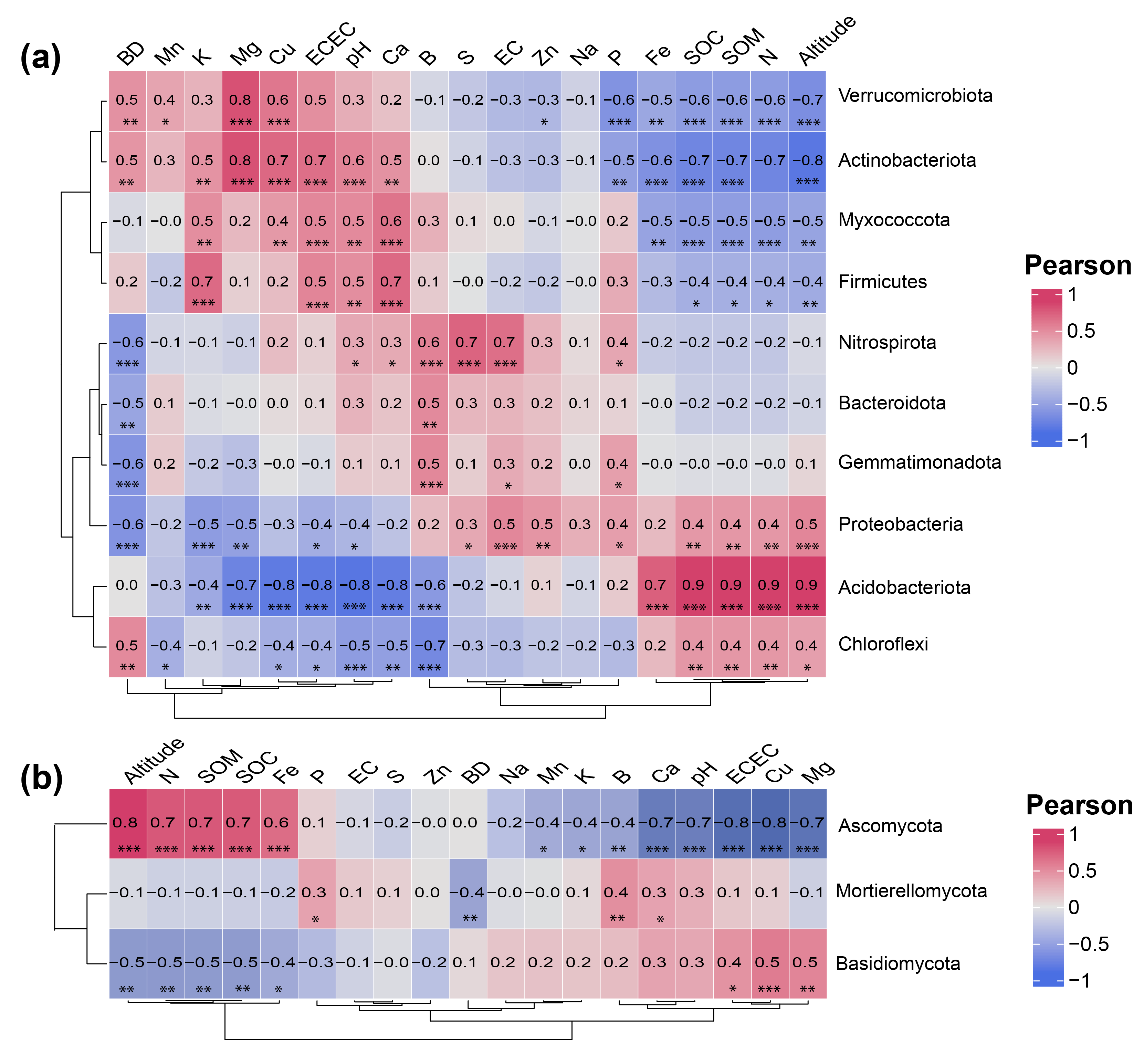

Supplement: Supplementary file 1 — ESM 1 [file 10123_2023_392_MOESM1_ESM.zip › Supplementary Figure S9.png]
